# Organ chip research in Europe: players, initiatives, and policies

**DOI:** 10.3389/fbioe.2023.1237561

**Published:** 2023-09-04

**Authors:** Renan Gonçalves Leonel da Silva, Alessandro Blasimme

**Affiliations:** Health Ethics and Policy Lab, Department of Health Sciences and Technology, ETH Zurich, Zurich, Switzerland

**Keywords:** organ chip, tissue chip, microphysiological systems (MPS), biomedical engineering, bioengineering, knowledge ecosystems, science policy, bibliometrics

## Abstract

**Background:** Organ chips are microfabricated devices containing living engineered organ substructures in a controlled microenvironment. Research on organ chips has increased considerably over the past two decades.

**Aim:** This paper offers an overview of the emerging knowledge ecosystem of organ chip research in Europe. Method: This study is based on queries and analyses undertaken through the bibliometric software Dimensions.ai.

**Results:** Organ chip research has been rapidly growing in Europe in recent years, supported by robust academic science consortia, public-private initiatives, dedicated funding, and science policy instruments. Our data shows that previous investment in basic and fundamental research in centers of excellence in bioengineering science and technology are relevant to future investment in organ chips. Moreover, organ chip research in Europe is characterized by collaborative infrastructures to promote convergence of scientific, technical, and clinical capabilities.

**Conclusion:** According to our study, the knowledge ecosystem of organ chip research in Europe has been growing sustainably. This growth is due to relevant institutional diversity, public-private initiatives, and ongoing research collaborations supported by robust funding schemes.

## 1 Introduction

Organ chips are miniature *in vitro* models of human organs created for biomedical research and drug discovery. Their aim is to mimic the functional components and characteristics of human organs and tissues, replicating the dynamic behavior, functionality, and pathophysiological responses of a living organism (Mummery et al., 2016; Sauer and Howard, 2018). They are manufactured at microscale and enable real-time monitoring (Mastrangeli et al., 2019a). The design of organ chips is carefully tailored to recapitulate the physiological characteristics of human organs, including specific cell types, their ratios, and the culture conditions needed to maintain viability (Huh et al., 2010; Kim and Takayama, 2015). Adult stem cells, primary patient cells, or commercially available cell lines can be used to develop organ-specific chips. Beyond offering models to study organ physiology, organ chip research allows scientists to overcome the limitations of using animal models to analyze drug response (van der Meer and van den Berg, 2020; Horejs, 2021). It is relevant to mention that the production of organ chips is a technically complex undertaking, and processes and technologies in this field continue to evolve at a considerable pace (Strelez et al., 2023). Moreover, organ chip research is a multidisciplinary domain merging biology, physiology, engineering, and microfabrication techniques. Each discipline contributes to the successful creation of microfluidic devices that mimic the structure and function of human organs, allowing researchers to study complex physiological processes and diseases in a controlled laboratory setting. The integration of different forms of expertise is key to the further development of the field. At the same time, organ chip research draws on consolidated advances in the field of tissue engineering, such as scaffold design and the experimental control of cell signaling and biomaterial interaction (Ahmed and Teixeira, 2022; Leung et al., 2022).

Technical questions about the development of organ chips have attracted attention from scientists, bioengineers, and technology developers from academia and industry. A growing number of events and conferences worldwide focus on advancing the state of the art of design and manufacturing of complex microphysiological systems, and connecting organizations in order to foster the introduction of organ chips as suitable animal substitutes for clinical trials. In Europe, the 2nd Annual Microphysiological Systems World Summit (Berlin, June 26–30, 2023) and the 3rd Next Gen Organ-on-Chip and Organoids workshop (Technopark Zurich, August 24–25, 2023) are examples of events that gather the community to discuss ways to accelerate the translation of advanced *in vitro* models in clinical and drug development. Additionally, they aim to expand upon action plans to address barriers associated with the adoption of new methods and technologies in a regulated environment (Cruelty Free Europe, 2023).

This paper explores the social and regulatory aspects of organ chip knowledge ecosystems in Europe. Due to the fast-rising number of global players in this field in the United States and Asia, it is critical to know how European organizations are positioned, and the types of policies and initiatives that could promote this field in the coming years.

## 2 Methods

This study offers an empirically grounded analysis of the knowledge ecosystem of organ chips in Europe. The concept of “knowledge ecosystems” is employed in science and technology studies to describe how scientific actors, funders, societal stakeholders, and regulators form communities of practice around specific forms of knowledge, with the aim of promoting, channeling, and regulating scientific activities in that area (Järvi et al., 2018). Knowledge ecosystems typically take the form of research networks and scientific infrastructure, composed of public and private research centers, consortia, civil society organizations, science policy actors, regulators, and firms, all collaborating to produce new knowledge and technologies (Järvi et al., 2018; da Silva et al., 2021). Increasingly, knowledge ecosystems are comprised of actors across different geographies and are typically formed in the early stages of research and development, prior to the competitive phases of innovation and commercialization.

Dimensions.ai is a scientific research database facilitating the exploration of research grant repositories, publications, clinical trials, patents, and policy documents. Dimensions.ai aggregates data from a variety of bibliographic repositories that are widely in use in academia, offering a powerful interface to customize and visualize results, thus facilitating data extraction and interpretation. The ability of Dimensions.ai to retrieve information from diverse data sources and explore how data are connected enables a broader and more insightful picture of scientific trends than is available from other scientific databases, making it well-suited to obtain a preliminary overview of an emerging knowledge ecosystem. There are similar tools available to run bibliometric analyses or visualize trends in academic publications, patent filings, and clinical trials registrations, e.g., VOSviewer, CiteSpace, and Netdraw (which extract data from publications in PubMed, Scopus, Web of Science), Orbit Intelligence and PatSeer (patents), and from Clinical Trials.gov and other repositories maintained by the National Institutes of Health of the United States (for information and results from ongoing or concluded clinical trials).

We used Dimensions.ai (Digital Science, 2023) to explore the organ chip knowledge ecosystem taking shape in five European countries (Germany, Netherlands, United Kingdom, Italy, and Switzerland). This selection represents the top five countries in Europe by number of publications about organ chips (Germany: 334; United Kingdom: 225; Netherlands: 155; Switzerland: 115; and Italy: 105). To our knowledge, Dimensions.ai is unique in combining multiple data sources from academic research organizations and commercial entities in the same platform (along with data from policy documents, national and transnational grant repositories, and publications in preprint). It improves analytical capacity by facilitating understanding of the knowledge ecosystem as a multi-disciplinary, multi-sectoral, and interconnected landscape, to an extent not possible with individual data queries. With the implementation of a robust automated system on this platform, which is continuously being tested by the platform’s staff to extract reliable data from official sources, the authors are confident that the accessed information maintains a high level of trustworthiness and accuracy.

While Dimensions.ai is an effective tool for analyzing research data in an integrated manner, and providing an overview of key players in a given scientific domain, it has some limitations. While this database provides access to verified research data, coverage, timeliness, and quality of the data may not be complete for all academic domains, sub-fields, or themes. For the time being, the tool provides only partial access to research data from private sector R&D activities. It should also be noted that Dimensions.ai, like similar tools, evolves continually to include more data sources and analytic features.

We explored the knowledge ecosystem of organ chip innovation in Europe along four analytic dimensions: publications trends, research organizations, research funding, and policy trends. In the results section, we illustrate our findings for each dimension.

We collected data on Dimensions.ai by searching for documents through a thematic query string (“organ-on-a-chip*” OR “organ chip*” OR “tissue chip*” OR “microphysiological systems”); we limited our search to the last 20 years (2003–2022). In total, we retrieved 18,654 publications and 676 grants (search conducted on 20 February 2023). Given the exploratory nature of the present study, we did not use exclusion criteria to screen our results, but subsequently focused our attention on the above-mentioned five countries. With this geographical restriction, we retrieved for the same period 3,991 publications (3,398 articles, 399 book chapters, 146 preprints, 39 conference proceedings, 11 books, and 111 grants). More than two-thirds (67.7%, N = 2,707) of the retrieved publications were published in open access.

We analyzed this data to understand current publication trends in a country-specific manner.

To understand which research organizations are most active in the space of organ chip research, we applied filters to produce a ranking of European research institutions with the highest number of publications in the field.

Dimensions.ai also enabled us to extract information about research funding agencies supporting organ chip research, and to collect relevant policy documents. Data on initiatives and regulations were extracted manually between March and May 2023, through literature review, reading of policy documents, and consultation of key funding agency websites, the European Commission and national medical agencies from each of the five selected countries.

Our method has limitations. As an exploratory study intended to capture a field overview of organ chip research initiatives in Europe, the method targeted collection and analysis of data on the general characteristics of the knowledge ecosystem. As result, initiatives in countries not ranked highest by number of publication, and recent projects and consortia that have not yet produced scientific publications, are not included. Despite such limitations, our study offers valuable insight into the innovation landscape surrounding organ chip research in Europe. This study can thus contribute to a clearer understanding of the knowledge ecosystem of organ chip research and help identify key player in this domain.

## 3 Results

### 3.1 Publication trends

**FIGURE 1:**
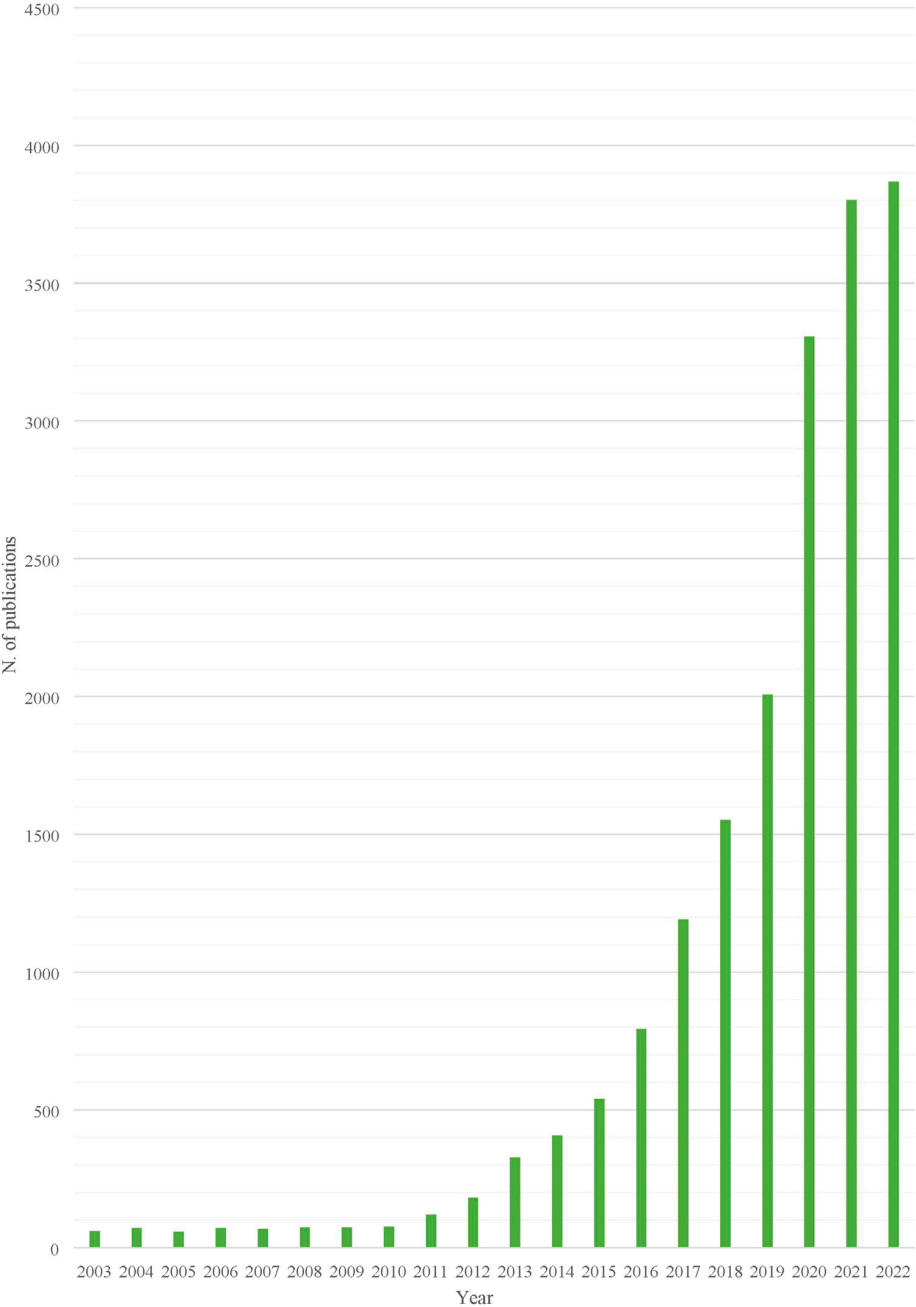
Yearly publications on Organ Chips (World, Total), 2003–2022.

For the last decade, publications in the field of tissue or organ chips have burgeoned globally (Figure 1). The United States leads by number of publications (*n* = 5,797), followed by China (*n* = 3,028). In the European context, publications are most prevalently produced in Germany (*n* = 1,453), the United Kingdom (*n* = 1,233), the Netherlands (*n* = 814), Italy (*n* = 762) and Switzerland (*n* = 582). Information about yearly publications by country is available in Figure 1.

**FIGURE 2:**
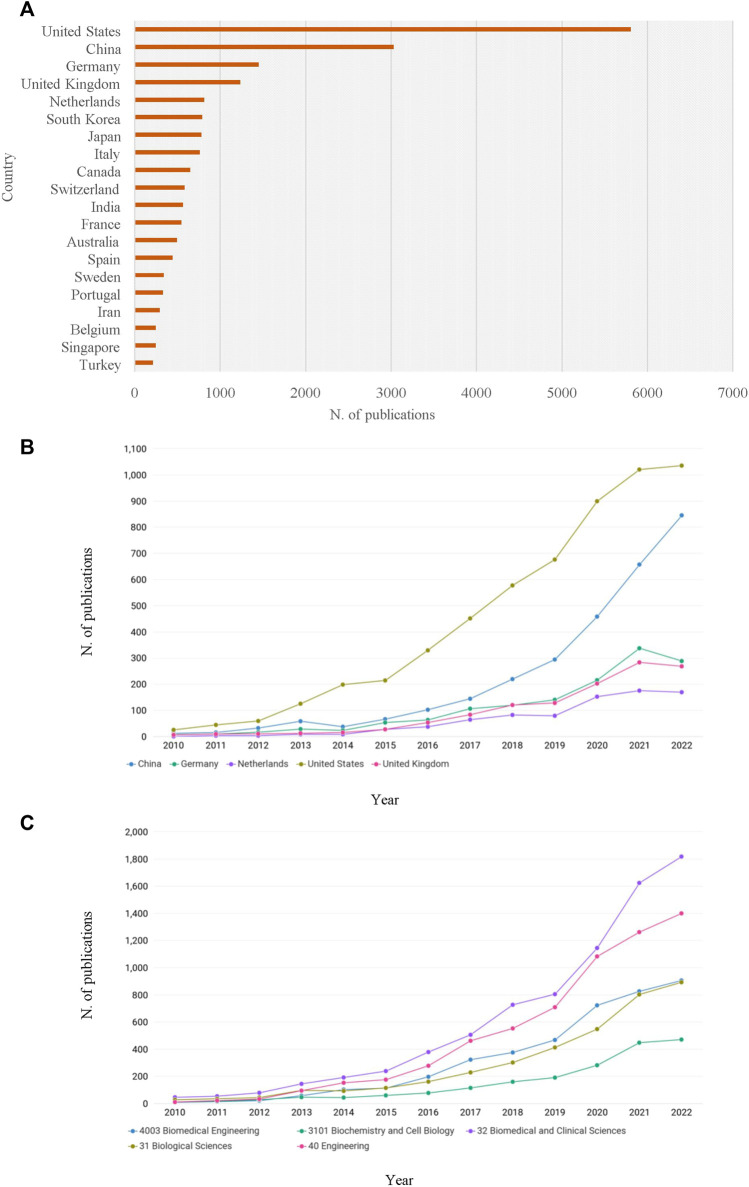
Publications, selected countries (Total, Timeline and Research categories) 2010–2022.

From a disciplinary perspective, publications were most frequently classified as “Biomedical and Clinical Sciences”, “Engineering”, “Biological sciences”, “Biomedical engineering”, “Medical Biotechnology”, or a combination thereof. (Figure 2C). While the rate of publication in the domain of organ chip research was relatively low up until 2010 (fewer than 20 publications annually), it began to grow steadily over the last decade, from 60 publications in 2014 to 919 in 2021, with a small decrease in 2022 (*n* = 814). The growth in the number of publications in selected European countries from 2010 is available in Figure 2B.

Globally, Harvard University is the leading research organization with 640 publications, followed by the Massachusetts Institute of Technology (*n* = 293). These figures may reflect pioneering work at Harvard’s Wyss Institute, where the first organ chip was developed in 2010 (Huh et al., 2010). In Europe, the most active research institution in the field is the University of Twente with 132 publications, followed by Utrecht University (*n* = 121), Karolinska Institute (*n* = 120), Leiden University Medical Center (*n* = 107), and the University of Minho (*n* = 106) (Figure 2A).

European research organizations have made significant contributions to organ chip research in recent years. In Germany, the University of Tübingen, University Konstanz, and the Technical University of Munich are the top-ranked research organizations in terms of the number of publications, with 85, 76, and 68 publications, respectively. The Technical University of Berlin and the Technical University of Dresden follow closely behind with 64 and 52 publications, respectively.

In the United Kingdom, the majority of organ chips publications are housed in research organizations located in the British Golden Triangle, specifically the University College London, AstraZeneca Headquarters, and Imperial College London, with 100, 89, and 89 publications respectively. The University of Cambridge follows with 79 publications.

In the Netherlands, the University of Twente, Utrecht University, and Leiden University Medical Center are the top-ranked research organizations in terms of the number of publications, with 132, 121, and 107 publications, respectively. The University Medical Center Utrecht and the University of Maastricht are also active in the field, with 81 and 78 publications, respectively. Additionally, the company Mimetas, responsible for the development of an early successful organ chip, has 61 publications.

In Italy, Politecnico di Milano, the European Commission’s Joint Research Centre, and the National Research Council hold the highest number of publications, with 84, 61, and 48 publications, respectively. The Italian Institute of Technology and the University of Milan follow closely behind with 47 publications each.

In Switzerland, the Institutes of Technology (ETH Zurich and EPFL) and Roche are at the forefront of organ chip research, with 102, 59, and 87 publications, respectively. The University of Basel and the University of Zurich are also central figures in the national knowledge landscape, with 43 and 39 publications, respectively.

**FIGURE 3:**
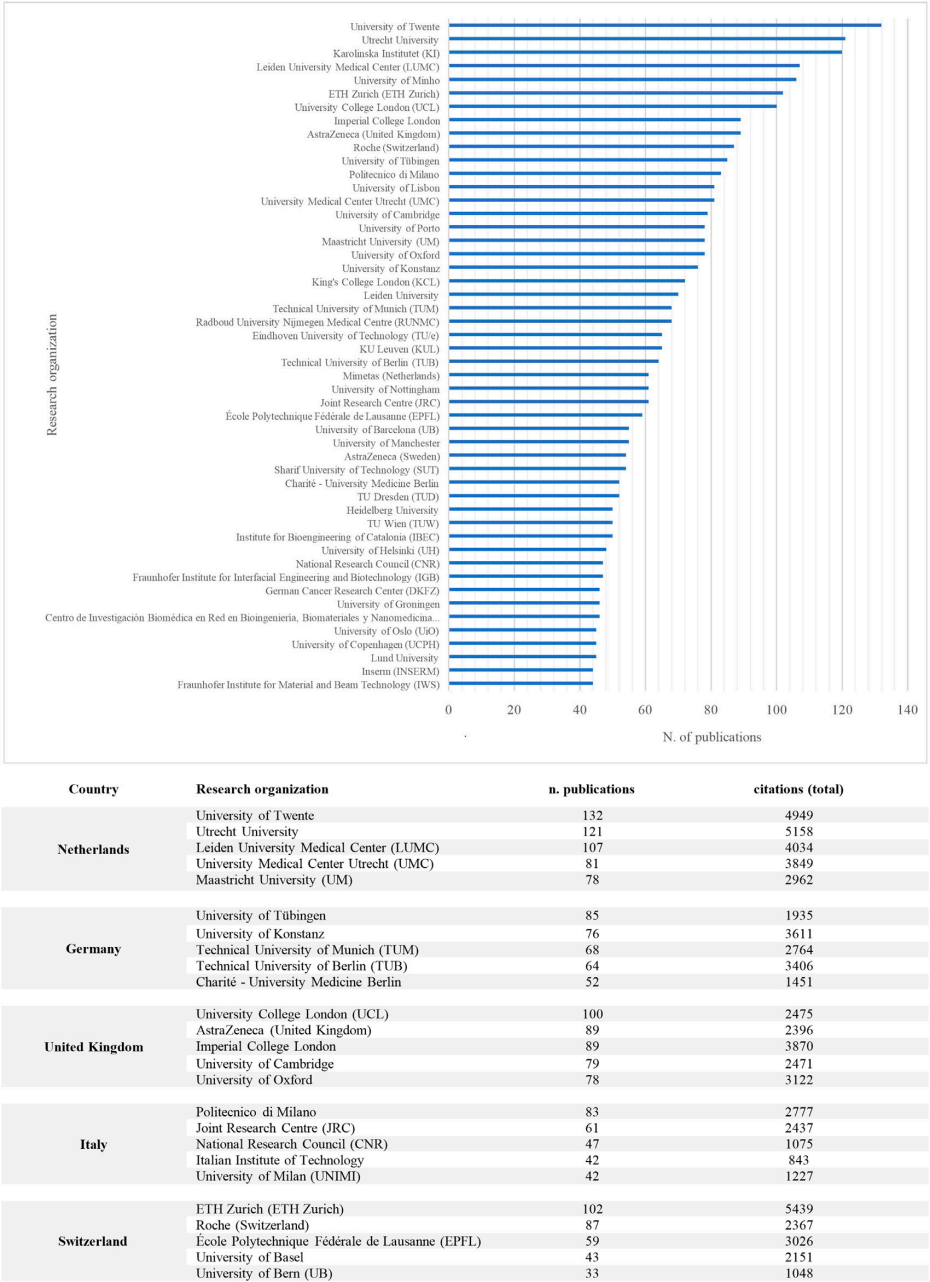
Number of publications by research organization (Top-50, selected Countries), 2003–2022.

Figure 3 presents a list of top-ranked research organizations by number of organ chip publications in the five selected countries.

### 3.2 Research collaborations

**FIGURE 4:**
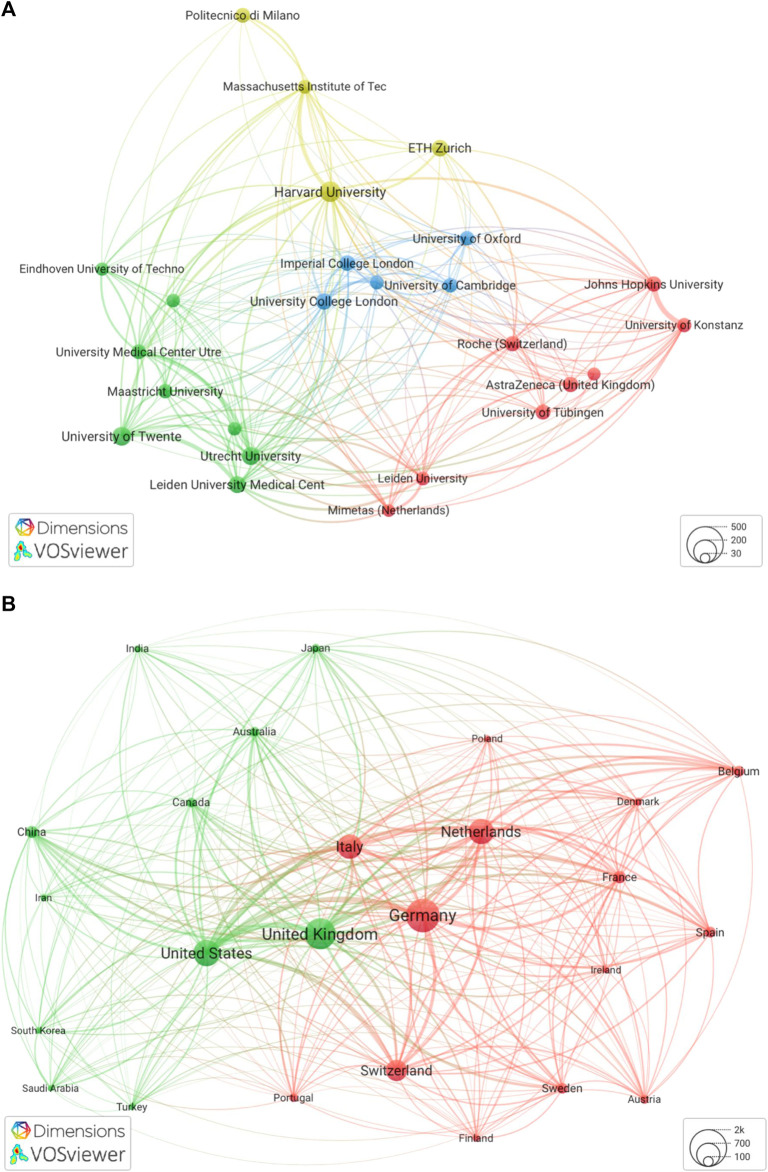
Network of research collaborations, selected organizations and countries (2003–2022)

We studied the landscape of research collaborations among scientists active in our subset of European countries. Co-authorship analysis was limited to 25 research organizations, to illuminate key players and allow for visualization of collaborations and clusters (Figure 4). Figure 4A illustrates the four main clusters (see Figure 4A). Two clusters are more geographically homogeneous, in the Netherlands (green) and the United Kingdom (blue); the remaining two clusters are more international, evidencing collaboration with research centers outside a specific geographic area (see yellow cluster featuring Harvard and MIT) as well as the presence of pharmaceutical partners (see red cluster featuring Roche and Astra Zeneca).

When looking at geographical relatedness in co-authored publications, we identified the existence of two clusters (see Figure 4B). One cluster (red) is composed of mostly European countries, with Germany, the Netherlands, Italy, and Switzerland most strongly represented; the other cluster (green) illustrates the United Kingdom as a major node, but includes mostly non-European countries (the US being the other major node).

### 3.3 Research consortia

Research efforts in Europe are often organized through consortia. Such initiatives focus on bringing together key players and improving harmonization of technical and experimental standards in the field of organ chip research.

Multiple initiatives have launched over the last decade to promote the successful integration of organ chip technologies in the European biomedical research infrastructure. Two examples are the Organ-on-Chip Development Project (ORCHID) and the Europe Organ-on-chip Society (EUROoCS). ORCHID is an EU initiative, coordinated by Leiden University Medical Center and the Dutch Organ-on-Chip consortium, hDMT. This project received funding from the European Parliament, 2021b research and innovation program (grant n. 766884). The initiative (2017–2019) sought to create a roadmap for organ chip technology development, along with a stakeholder network (ORCHID, 2023).

Likewise, EUROoCS, established in 2018 as a not-for-profit organization, continued many of the efforts of ORCHID, bringing together organ chip scientists, industry, and government regulators in support of research and development (European Organ-on-Chip Society, 2022). Similar to the US context, the EUROoCS has prioritized standardization. In creating their priorities, EUROoCS referenced the success of the National Center for Advancing Translational Sciences (NCATS) (2022) at the National Institutes of Health (NIH), in funding the development of multiple organ chip models, and conducting the external testing and standardization requisite for market acceptance and integration.

Indeed, many policy reports published by the European Commission and other European agencies refer to the success of organ chip models in the US as an example to follow in terms of the knowledge ecosystem that has been established there. In December 2022, the “FDA Modernization Act 2.0.” was signed into law by the Biden-Harris administration, representing a major shift in the regulatory landscape that paves the way for innovative modeling approaches in early stages of drug discovery and innovation. Following years of advocacy, the bill officially authorizes the use of alternatives to non-human animal models in pre-clinical pharmaceutical testing. The bill points to cell-based assays, predictive computer models, and organ chips as examples of technologies that can be used in place of non-human animal models, which have long been required by the FDA. With the passage of this bill, animal studies are no longer required in pre-clinical testing whenever an alternative suitable method is available to demonstrate drug safety and efficacy of therapeutic candidates and products (Bill S.5002).

Our study accessed data about multiple national initiatives in the field, described in Table 1 (focused on the top five countries by number of scientific publications).

**TABLE 1:** Selected initiatives on organ chips research, selected countries, 2003–2022.

Year	Initiative	Anacronym	Country of headquarters	Project aims
2017 - current (until 2027)	Netherlands Organ-on-Chip Initiative Project	NOCI	Netherlands	Netherlands Organ-on-Chip Initiative (NOCI) has been awarded a prestigious NWO Gravitation subsidy (Zwaartekracht premie) of 18.8 million euros. NOCI aims at creating a new platform, based on a combination of human stem cells and microchips, to learn more about the development of diseases and to better predict the effect of medicines, and will be a decisive step towards personalized healthcare. (Netherlands Organ-on-Chip Initiative, 2023)
2015–current	Institute for human organ and Disease Model Technologies	hDMT		The Institute for human organ and Disease Model Technologies (hDMT) is a consortium consisting of 14 partner organizations, including technical universities, university medical centers and knowledge institutes. It brings together researchers from different disciplines, varying from technologists and biologists to pharmacologists and clinicians (hDMT, 2023)
2017–2019	Organ-on-chip in development	ORCHID		The Organ-on-Chip development project (ORCHID) is an EU initiative, coordinated by Leiden University Medical Center and the Dutch Organ-on-Chip consortium hDMT. ORCHID aims to create a roadmap for organ-on-chip technology and to build a network of all relevant stakeholders in this field (ORCHID, 2023)
2018–current	European Organ-on-chip Society	EURoOCs		The European Organ-on-chip Society (EURoOCs) is an independent not-for-profit organization aimed at encouraging Organ-on-Chip research and development, to share and advance knowledge and expertise in the field (EUROoCS, 2023)
2021–current	SMART Organ-on-chip Project	SMART-OoC		The SMART Organ-on-chip Project aims to “develop and integrate 1) a standardized microfluidic SMART docking plate into which chip modules can be plugged
				2) technical chip modules for microfluidic actuation and sensing
				3) readout technologies for multiparameter monitoring; and 4) prototype tissue chip modules with 3D organ architectures and integrated tissue microenvironment (…)
				5) demonstrate functionality of the SMART OoC models by inducing inflammation and testing drugs (SMART OoC, 2023)
2023–current	Organ-on-Chip Centre Twente	OoCCT		The Organ-on-Chip Centre Twente (OoCCT) is a centre of expertise supported by the MESA + Institute and the TechMed Centre of the University of Twente. OoCCT aims to provide services to researchers and companies outside of the University of Twente and give them access to the technology in order to accelerate innovation and real-world application of Organs-on-Chips (University of Twente, 2023b)
2018–2022	Organ-on-a-chip Technology Network	OCTN	United Kingdom	The United Kingdom Organ-on-a-Chip Technologies Network (OCTN) was established in 2018 to represent the United Kingdom community of scientists, industrialists, clinicians, funders and regulators working in the area of organ-on-a-chip technology (University of Twente, 2023a)
2020 - current	Centre for Predictive *in Vitro* Models (QM + Emulate Centre) at Queen Mary University of London	OCI/QMU		The CPM brings together academics developing and using predictive *in vitro* models across the faculties of Science and Engineering and Medicine and Dentistry at Queen Mary University of London (QMUL Emulate Centre, 2023)
2021 - current	The Wellcome Leap Health Breakthrough Network - Human Organs, Physiology and Engineering HOPE Program	HOPE		The HOPE Program “aim to leverage the power of bioengineering to advance stem cells, organoids, and whole organ systems and connections that recapitulate human physiology *in vitro* and restore vital functions *in vivo*.” According to its official website (HOPE Program, 2023) the program has two key goals: “1. Bioengineer a multiorgan platform that recreates human immunological responses with sufficient fidelity to double the predictive value of a preclinical trial with respect to efficacy, toxicity and immunogenicity for therapeutic interventions targeting cancer, autoimmune and infectious diseases, and 2. Demonstrate the advances necessary to restore organ functions using cultivated organs or biological/synthetic hybrid systems.”
(2016) 2021 - current	MicroOrganoLab Tubingen	OC Tubingen	Germany	The initiative µOrganoLab brings basic and translational research and people from different disciplines working together to develop Organ-on-Chip systems as well as enabling technologies to better understand human biology (Micro Organo Lab Tubingen, 2023)
2021–current	Organ-on-a-chip Working Group - Natural and Medical Sciences Institute	NMI Organ-on-a-chip		The Organ-on-a-chip Working Group of the Natural and Medical Sciences Institute of Reutlingen, Germany (NMI Naturwissenschaftliches und Medizinisches Institut) is an interdisciplinary team working at the interface between material and engineering sciences, physics, biology and medicine. The initiative is driven by the principle of reducing use and necessity of animal testing according to the 3R principle (Replace, Reduce, Refine), as well as “to increase the transferability of preclinical results to the clinical phases and thus to make the entire development more cost-effective, safer and faster.” (NMI Organ-on-a-chip)
2021 - current	3R-Center Tübingen for *In vitro* Models and Alternatives to Animal Testing	3R Tubingen		The 3R Tubingen Center for *in vitro* Models and Alternatives to Animal Testing (3R Tubingen) aims to create a broad, interdisciplinary awareness of 3R approach with a focus on “Replacement”, working on the development of a technology platform for own development or to the 3R-Network Baden-Württemberg partners. (3R Tubingen, 2023)
2020 - current	Organ Chips Group German Cancer Research Center	Orgn Chips DKFZ		The Epithelium Microenvironment Interaction Laboratory is a research group based in the German Cancer Research Center in Heidelberg, and it is specialized in developing and manipulation of human organoid and organ-on-a-chip models to study the roles of bacteria, immune cells and other microenvironmental factors in cancer. The group takes place in the research division Microbiome and Cancer, a bridging division between DKFZ Heidelberg and the Weizmann Institute of Science of Israel
2022 - now	Società Italiana Organ-on-Chip	SIOoC	Italy	The Italian Organ-on-Chip Society (SIOoC) was founded in 2022 aiming to become a meeting point for Italian researchers working in the field of organ-on-chip development. According to its website, “SIOoC promotes advanced training and scientific dissemination on the issue of organ-on-chip. It also promotes dialogue with stakeholders (companies, institutions, regulatory bodies), also through the establishment of thematic tables.” (SIOoC, 2022)

### 3.4 Funding

National and European funding agencies play a key role in shaping the organ chip landscape in Europe. Our search enabled us to extract information about funders appearing in the acknowledgment section of publications in the field. Figure 4 ranks the top twenty funding agencies in the field by frequency of acknowledgement. The leading funding agencies (ranked by number of publications resulting from grants) are the European Commission (EC) (*n* = 16 grants; Aggregate funding amount: USD 46.8 M); the European Research Council (ERC) (*n* = 4 grants; USD 4.8 M); and the German Federal Ministry of Education and Research (BMBF) (*n* = 16 grants; USD 9.7M). The research organizations that received more grants from EC and ERC (*n* = 39 grants combined) were three nascent biotechnology companies, Eveflow (France, *n* = 6, value in US$ 10.4 mi/EUR 9.27 mi), Mimetas (Netherlands, *n* = 4, US$ 15.4 mi/EUR 13.72 mi), and Cherry Biotech (France, *n* = 4, US$ 4.1 mi/EUR 3.65 mi). National funding instruments like the German Research Foundation (Federal Ministry of Education, *n* = 16, US$ 9.7 mi/EUR 8.64 mi) and the “Engineering and Physical Research Council” of the United Kingdom (EPRSC, *n* = 27, US$ 2.3 mi/EUR 2.05 mi) have funded projects in public institutes, technical universities, and medical centers, such as the Fraunhofer Society (*n* = 2, US$ 1.5 mi/EUR 1.34 mi) and Technical University of Berlin in Germany, and the University of Southampton, in partnership with international collaborators from the Max Planck Society (*n* = 2, US$ 1.6 mi/EUR 1.43 mi). Results about R&D expenditures, including data about countries and funding agencies (extracted from “Location of research organization/Grants”: n = 111/Analytical views: Funders), are available in Table 2.

**TABLE 2:** R&D Expenditure by funding agencies (Grants), Selected countries, 2003–2022.

Location of research organization (country)	Funder/Agency	Country	Number of grants	Funding amount (aggregated, in Euros)
Germany	European Commission (EC)	EC	8	28.151.392,00
	Federal Ministry of Education and Research (BMBF)	Germany	16	8.621.578,00
	European Research Council (ERC)	EC	1	1.918.038,00
	Engineering and Physical Sciences Research Council (EPSRC)	United Kingdom	1	1.332.319,00
	Medical Research Council (MRC)	United Kingdom	1	336.586,00
	Deutsche Forschungsgemeinschaft (DFG)	Germany	5	information not available
	Volkswagen Foundation (VolkswagenStiftung)	Germany	2	information not available
	German Association of Joint Industrial Applied Research Institutes (AIF)	Germany	1	information not available
United Kingdom	European Commission (EC)	EC	8	29.924.923,63
	Engineering and Physical Sciences Research Council (EPSRC)	United Kingdom	27	1.963.592,45
	Medical Research Council (MRC)	United Kingdom	7	1.482.581,54
	Biotechnology and Biological Sciences Research Council (BBSRC)	United Kingdom	7	1.188.159,13
	Swiss National Science Foundation (SNF)	Switzerland	1	680.649,29
	Innovate United Kingdom (Innovate United Kingdom)	United Kingdom	2	426.914,41
	Wellcome Trust (WT)	United Kingdom	1	340.075,60
	National Centre for the Replacement Refinement and Reduction of Animals in Research (NC3Rs)	United Kingdom	3	194.618,37
	Deutsche Forschungsgemeinschaft (DFG)	Germany	1	information not available
	National Research Council (CNR)	Italy	1	information not available
Netherlands	European Commission (EC)	EC	8	19.384.162,38
	European Research Council (ERC)	EC	2	2.078.299,69
	Medical Research Council (MRC)	United Kingdom	1	336.586,37
	Dutch Research Council (NWO)	Netherlands	4	information not available
	Netherlands Organisation for Health Research and Development (ZonMw)	Netherlands	6	information not available
Switzerland	European Commission (EC)	EC	4	10.817.940,65
	Swiss National Science Foundation (SNF)	Switzerland	5	3.473.624,54
	Engineering and Physical Sciences Research Council (EPSRC)	United Kingdom	1	information not available
Italy	European Commission (EC)	EC	3	12.253.602,86
	European Research Council (ERC)	EC	1	153.551,26
	Italian Association for Cancer Research (AIRC)	Italy	2	information not available
	National Research Council (CNR)	Italy	1	information not available

^a^ Data displayed in this table does not capture the precise values of R&D expenditure on Organ Chips by country and funding agency. The purpose of this table is, then, serve as reference to what agencies and countries can be highlighted based in data automatically extracted and available in dimensions.ai.

^b^ Values were changed from US$ to Euros (1 US$ = 0.89 Euros).

Source: Elaborated by the authors with data from Dimensions.ai. (Digital Sciences)

## 4 Discussion

Our study highlights the significance of institutional diversity, research collaborations, and public-private initiatives that promote organ chip research in Europe, as well as the role of public funding in supporting the knowledge ecosystem in this field.

According to a study by da Silva et al. (2020), R&D initiatives are influenced by cultural and political factors. Our study shows the utility of novel bibliometric tools such as Dimensions.ai to reconstruct emerging knowledge ecosystems. This approach can greatly contribute to the understanding of scientific research practices and inform science policy activities to stimulate innovation in many countries - especially in emerging sectors of biotechnology (Karaulova et al., 2016; Au and da Silva, 2021; Heimeriks and Boschma, 2013; Partelow et al., 2020; da Silva et al., 2023).

Regulation is certainly one of the key political factors affecting innovation. Evens and Kaitin (2015) observe that early regulatory reforms and the standardization of national legal frameworks for research involving bioengineered systems and tools have significantly influenced the evolution and pathways of bioengineering research and innovation in Europe over past decades (Ewart, 2022).

Research on the role of policies and regulations in the context of organ chip research has so far been limited (Kemp et al., 2020). However, it is possible to observe a close association between the development of this field and the legal framework designed to reduce the use of non-human animals in scientific research (Brackenbury, 2017). Similar associations have been identified in the United States, as noted by Heringa et al. (2020) and da Silva and Blasimme (2023).

In Europe, the explicit effort to foster the development of new microphysiological systems and other bioengineered alternatives to animal research began with the enactment of DIRECTIVE 2010/63/EU on 22 September 2010 (European Commission, 2010). This directive endorses the use of alternative methods to animal testing whenever possible, allowing animal testing only as a last resort when no other suitable method is available (Alternatives to Animal Experimentation ALTEX, 2018; National Center for Advancing Translational Sciences, 2022).

Subsequently, the EU has established a regulatory framework for the utilization of alternative methods to animal testing, which encompasses the validation and acceptance of these methods (Moraes et al., 2013; van Meer et al., 2017; Mastrangeli et al., 2019; Politico, 2021).

As mentioned in Table 2, in September 2021, the European Parliament approved a resolution to stimulate EU members to adopt a strategic plan with “ambitious and achievable objectives and timelines for transitioning to a research system that does not rely on animals for research and testing.” (European Parliament, 2021a). While not legally binding, the resolution clearly indicates a policy direction for the European Union and invites further legislative activity that is likely to create incentives for organ chip research in Europe (Human Society International, 2021).

Researchers, investors, and regulators share the belief that human organ chips hold great potential for replacing animal models in drug development and serving as living avatars for personalized medicine. According to Nahle (2022), organ chip technology can be seamlessly integrated into the drug development pipeline, from early drug discovery to preclinical stages. This paradigm shift could lead to a post-animal testing era in drug discovery (Wyss Institute, 2014; Herpers, 2022; Ingber, 2022; Zainzinger, 2022).

Key regulations addressing organ chip research and development activities are available in Table 3.

**TABLE 3:** Selected regulations on organ chips research, EU level and selected countries, 2003–2022.

Country	Year	Regulation	Anacronym	About
European Union	2007	Regulation on Advanced Therapy Medicinal Products	ATMP Regulation 1394/2007	This Regulation introduces additional provisions to those laid down in Directive 2001/83/EC. It regulates advanced therapy medicinal products which are intended to be placed on the market in Member States and either prepared industrially or manufactured by a method involving an industrial process (European Parliament, 2007)
	2009	EU Regulation 1223/2009	EU Regulation 1223/2009	It covers the safety and efficacy of cosmetic products and requires that cosmetic products be tested in animal models before they can be sold in the EU, and tissue chips can be used as an alternative to animal testing for this purpose (European Parliament, 2009)
	2010	DIRECTIVE 2010/63/EU	DIRECTIVE 2010/63/EU of 22 September 2010	Considered the first explicit move towards new microphysiological systems and other bioengineered alternatives to animal research. This directive requires the use alternative methods to animal testing whenever possible; animal testing is only permitted when no other suitable method is available (European Commission, 2010)
	2017	*In vitro* Diagnostic Medical Devices Regulation	IVDR Regulation (EU) 2017/746	The *In Vitro* Diagnostic Medical Devices Regulation (EU) 2017/746 (IVDR) establishes a new regulatory framework for *in vitro* diagnostic medical devices. It is estimated that around 70% of clinical decisions are made using *in vitro* diagnostic medical devices (European Parliament, 2017)
	2021	European Parliament Resolution on plans and actions to accelerate a transition to innovation without the use of animals in research, regulatory testing and education	2021/2784 (RSP)	The resolution recalls the objectives of the Directive on the protection of animals used for scientific purposes (2010/63/EU) and the replacement of procedures on live animals as soon as it is scientifically possible. It requires the European Commission to establish an inter-service taskforce, including Member States and agencies, to develop action plans to accelerate the development of the alternative animal-free methods, technologies and instruments, and to address implementation and enforcement issues. (European Parliament, 2021a)
United Kingdom	1986 (Amended multiple times)	Animals (Scientific Procedures) Act 1986	THE ANIMALS ACT of 1986	The Animals (Scientific Procedures) Act of 1986 regulates the use of animals in scientific research, including the development and testing including disease models and bioengineered tissues. It requires that animal testing be avoided wherever possible and that alternatives to animal testing be used where available
	2003	The Medicines and Healthcare Products Regulatory Agency	MHRA	The Medicines and Healthcare Products Regulatory Agency (MHRA) was created in 2003 and is responsible for regulating the use of medical devices in the United Kingdom, including tissue chips. Tissue chips that are intended for use as medical devices must meet the safety and performance requirements set out by this agency (MHRA, 2023)
	2004	The National Centre for the Replacement, Refinement, and Reduction of Animals in Research	NC3Rs	The National Centre for the Replacement, Refinement, and Reduction of Animals in Research (NC3Rs) was created in 2004 in response to the House of Lords Select Committee report on Animals in Scientific Procedures. It is a UK-based organization that promotes the development and use of alternatives to animal testing. It provides guidance and support to researchers who wish to use tissue chips as an alternative to animal testing
	2004	Human Tissue Act	HUMAN TISSUE ACT of 2004	This act regulates the removal, storage, use and disposal of human tissue, including tissue chips or other microphysiological systems that may be derived from human tissue (Human Tissue Act, 2004)
Germany	1961 (Amended in 2019*)	German Medicines Act, The Drug Law	Arzneimittelgesetz, AMG	Act to guarantee safety in respect of the trade in medicinal products in the interest of furnishing both human beings and animals, ensuring in particular the quality, efficacy and safety of medicinal products. (German Medicines Act, 2019)
	1989 (Amended in 2018*)	German Medical Devices Act	Medizinproduktegesetz, MPG	This Act is to regulate the trade in medical devices and, by doing so, to guarantee the safety, suitability and performance levels of medical devices as well safe- guard the health and ensure the necessary protection of patients (German Medical Devices Act, 2018)
	2013	Act on the Protection of Animals Used for Scientific Purposes	Animal Welfare Act of 2013	This Act regulates the use of animals in scientific research, including the use of tissue chips or other microphysiological systems in animal testing. (Animal Welfare Act, 2013)
Netherlands	1999	Dutch Medical Research Involving Human Subjects Act	Wet medisch-wetenschappelijk onderzoek met mensen, WMO	The Dutch Animals Act of 1999 regulates the use of animals in scientific research, including to develop and test tissue chips. It requires that animal testing be avoided wherever possible, and that alternatives such as tissue chips be used instead
	2011	Dutch Animals Act	Wet dieren	Wer dieren attests that animal testing is only permissible when there is no suitable alternative and the purpose of the research outweighs any inconvenience to the animal
	2014	Act on the Use of Animals in Scientific Research	Wet op de Dierproeven	This Act regulates the use of animals in scientific research, including the use of tissue chips or other microphysiological systems in animal testing
Switzerland	2000	Federal Act on Medicinal Products and Medical Devices (Therapeutic Products Act)	SR 812.21 - Federal Act of 15/12/2000	This Act aims to protect human and animal health, and to guarantee that only high quality, safe and effective therapeutic products are placed on the market.
	2005	The Swiss Animal Welfare Act	SR 455 - Animal Welfare Act of 16/12/2005, AniWA	The Swiss Animal Welfare Act of 2005 sets out detailed regulations on animal husbandry and research with animals, and includes Tissue Chips as suitable animal substitute in respect to the 3R principle (Swiss Animal Welfare Act, 2005)
	2011	Federal Act on Research involving Human Beings	SR 810.30 Human Research Act, HRA	This act regulates the ethical and legal requirements for research involving human subjects, including the use of tissue chips or other microphysiological systems that may be derived from human tissue (Swiss Human Research Act, 2011)
Italy	2003	Legislative Decree no. 211/2003	Legislative Decree no. 211/2003	This Legislative Decree establishes specific provisions regarding the conduct of clinical trials, including multi-centre trials on human subjects involving medicinal products as defined in section 1 of Legislative Decree no. 178 of 29 May 1991
	2010	Law on Research and Innovation (240/2010)	Law on Research and Innovation (240/2010)	This law provides a framework for the organization and funding of scientific research, which may include the use of tissue chips or other microphysiological systems

Dimensions.ai is an effective tool for analyzing data in an integrated manner, and contributes significantly to studies aiming to provide an overview of key players of emerging knowledge ecosystems. The tool, however, is still under development, and has relevant limitations in terms of lacking access to precise data about R&D expenditures from national and supranational levels, or from industry. Dimensions.ai, then, should be taken as a complementary tool to support studies on knowledge ecosystems, gaining explanatory power when combined with multiple methods of data collection, analysis, and visualization.

## 5 Concluding remarks

Organ chip research has gained international recognition as a prominent area of biomedical engineering innovation in recent years. In Europe, the convergence of research efforts, funding, and regulatory incentives has shaped a robust knowledge ecosystem that places many European research institutions as key international players in the field. More research is needed to monitor whether and how, in coming years, present incentives will continue to promote innovation in organ chip research in the European context.

## Data Availability

The original contributions presented in the study are included in the article/Supplementary materials. Further inquiries can be directed to the corresponding author.
